# Mitigating Impacts of the COVID-19 Pandemic on Gorilla Conservation: Lessons From Bwindi Impenetrable Forest, Uganda

**DOI:** 10.3389/fpubh.2021.655175

**Published:** 2021-08-12

**Authors:** Gladys Kalema-Zikusoka, Stephen Rubanga, Alex Ngabirano, Lawrence Zikusoka

**Affiliations:** ^1^Conservation Through Public Health, Entebbe, Uganda; ^2^Gorilla Conservation Coffee, Entebbe, Uganda; ^3^Bwindi Development Network, Kanungu, Uganda

**Keywords:** COVID-19, gorillas, conservation, one health, livelihoods

## Abstract

The COVID-19 pandemic, affecting all countries, with millions of cases and deaths, and economic disruptions due to lockdowns, also threatens the health and conservation of endangered mountain gorillas. For example, increased poaching due to absence of tourism income, led to the killing on 1st June 2020 of a gorilla by a hungry community member hunting duiker and bush pigs. Conservation Through Public Health (CTPH), a grassroots NGO and non-profit founded in 2003 promotes biodiversity conservation by enabling people to co-exist with wildlife through integrated programs that improve animal health, community health, and livelihoods in and around Africa's protected areas and wildlife rich habitats. Through these programs, we have helped to mitigate these impacts. CTPH worked with Uganda Wildlife Authority and other NGOs to improve great ape viewing guidelines and prevent transmission of COVID-19 between people and gorillas. Park staff, Gorilla Guardians herding gorillas from community land to the park and Village Health and Conservation Teams were trained to put on protective face masks, enforce hand hygiene and a 10-meter great ape viewing distance. To reduce the communities' need to poach, CTPH found a UK-based distributor, for its Gorilla Conservation Coffee social enterprise enabling coffee farmers to earn revenue in the absence of tourism and provided fast growing seedlings to reduce hunger in vulnerable community members. Lessons learned show the need to support non-tourism dependent community livelihoods, and more responsible tourism to the great apes, which CTPH is advocating to governments, donors and tour companies through an Africa CSO Biodiversity Alliance policy brief.

## Introduction

The One Health approach recognizes that the health of people is closely connected to the health of animals, plants, and their shared environment (https://www.onehealthcommission.org/en/why_one_health/what_is_one_health/).

In November 2019, a highly contagious novel coronavirus, SARS-COV-2 closely related to bat coronaviruses was identified in China and some of the index cases linked to a wet live animal market in Wuhan, Hubei Province (1). COVID-19 affects both the upper respiratory and lower respiratory tracts with a mortality rate of as much as 6.5% of confirmed cases (2). With an estimated 80% of infected people becoming asymptomatic, a combination of increased urbanization (3), land use change (4), high human population growth rates and air travel (5) resulted in the virus spreading from one continent to another and within most countries in the world in a period of four months. World Health Organization (WHO) declared the COVID-19 epidemic as a pandemic on 11th March 2020.

By December 2020, there were over 80 million confirmed cases and 1.8 million deaths (Worldometer.com/coronavirus), and more infectious mutated strains and variants of the SARS-COV-2 virus were discovered resulting in additional lockdowns and travel restrictions. Though the African continent and Uganda in particular have had relatively few cases (6), the health care systems have been overwhelmed during the waves caused by a sharp rise in infections and deaths, in the few and inadequately equipped hospitals. Several cases and deaths among people in marginalized rural areas have gone undetected because people have inadequate health seeking behavior and majority cannot afford to pay for testing. These include people living in biodiversity hotspots that are rich in wildlife and have high human population densities.

Communities bordering protected areas in Africa are among the most marginalized with limited access to basic health and other social services as well as livelihoods options. Improving the well-being of communities bordering protected areas has contributed to conservation outcomes (7). Improving community health has the potential to reduce the risk of zoonotic disease transmission between people and great apes (8) and improve their attitudes to conservation (9). Improving community livelihoods has the potential to reduce hunger and the need to poach. Great apes are found within 21 countries in Africa of which 13 have great ape tourism at 33 sites. Ecotourism has provided benefits to local communities who are employed by locally based organizations to protect the wildlife or set up enterprises that sell crafts, food items, accommodation, and experiences including community walks and traditional entertainment, as well as services as porters who carry bags of tourists visiting gorillas and chimpanzees. This form of alternative livelihood has reduced the communities' dependence on the forest for food and fuel wood, contributing to the protection of endangered wildlife. Global lockdowns caused by the COVID-19 pandemic disrupted this sustainable financing for conservation by preventing travelers who provide critical revenue for conservation and sustainable development from reaching these tourist sites.

## Impact of COVID-19 on Gorilla Conservation

Humans and non-human great apes in Africa share over 98% DNA genetic material and zoonotic diseases have been transmitted between them causing morbidity and mortality (10–12). Furthermore, studies indicate that gorillas, chimpanzees, and other old world primates are just as susceptible as humans to COVID-19 because they have the same Angiotensin Converting Enzyme (ACE2) protein receptors that the SARS-COV2 virus attaches to (13, 14), making them highly susceptible to SARS-COV2 from humans. Wild great apes are at risk of contracting human diseases from the people they interact with including park staff, conservation personnel, researchers, tourists, and local communities. The first natural transmission of COVID-19 to primates occurred within 1 year of the pandemic in January 2021 when eight gorillas at San Diego Zoo Safari Park contracted the disease from an asymptomatic zoo keeper with three of them testing positive through fecal sample testing. A 48-year-old adult male gorilla developed severe signs and the rest of the younger members of the troop developed mild signs of COVID-19 (15, 16). Thus, the COVID-19 pandemic has not only resulted in a breakdown of human health care systems due to an overwhelming number of cases and disruptions to economies due to global lockdowns, but also presented a new threat to the conservation of wildlife. For a species as endangered as the mountain gorillas, the balance between health and economics has become even more critical for their survival during this pandemic (17).

**Figure 1:**
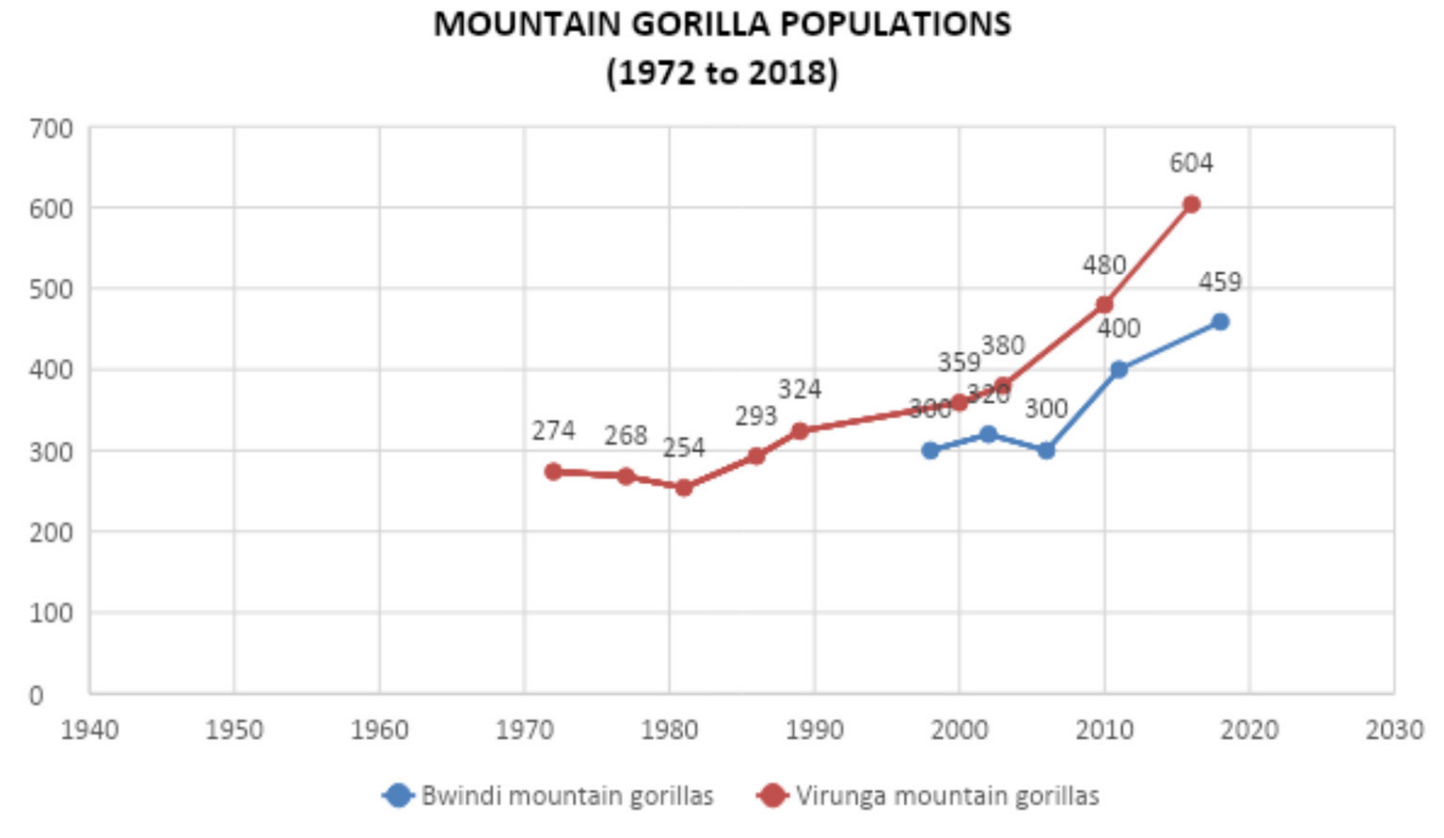
Mountain gorilla population census results from 1970 to 2020 (Source compiled by Conservation Through Public Health).

The loss of tourism income for local communities bordering protected areas in Africa, due to a reduction in the number of tourists whose presence also provides some protection for the wildlife contributed to an increase in poaching (18). This could reverse the trend and conservation gains brought about by tourism for endangered mountain gorillas whose IUCN status was downgraded from critically endangered in 2018 as the only gorilla sub species showing a positive growth trend (Figure 1) in their population (19). Within the first 3 months of the pandemic reaching Uganda in March 2020, there was at least a doubling of snares retrieved with the same level of patrol effort at protected areas in Uganda as stated by the Uganda Wildlife Authority (20) including Bwindi Impenetrable National Park (BINP), home to 43% of the world's estimated 1,063 mountain gorillas (21). The absence of tourism due to the COVID-19 pandemic contributed to the killing on 1st June 2020 of the lead silverback of the Nkuringo gorilla group in BINP by a hungry and vulnerable community member hunting duiker and bush pigs for food and sale at the local market. Gorillas are not eaten in Uganda, but become accidental victims of snares set for other species. When the poacher speared a bush pig, its scream prompted the silverback gorilla to charge him to protect his family. The poacher then speared and killed the gorilla, claiming that it was in self defense. This killing of an endangered gorilla, a direct result of COVID-19 generated a worldwide acknowledgment of the devastating impact of the pandemic on the conservation of wildlife. The poacher was sentenced to 11 years in jail, the longest that any person in Uganda has been sentenced for killing wild animals (22). Though this tough sentence was a deterrent to other community members, the increasing hunger due to the lack of tourism was likely to result in other similar incidents among desperate community members.

## Building a One Health Approach to Conservation

Conservation Through Public Health (CTPH), a grassroots Ugandan NGO and US registered non-profit founded in 2003 promotes biodiversity conservation by enabling people to co-exist with gorillas and other wildlife through integrated programs that improve animal health, community health and livelihoods in and around Africa's protected areas and wildlife rich habitats. CTPH was established following fatal scabies skin disease outbreaks in Bwindi mountain gorillas that were traced to local communities with less than adequate hygiene and health services (23, 24). Through previously established grassroots programs founded on the principles of One Health, PHE (Population, Health and Environment) by adding community based family planning (25) and Planetary Health (26), CTPH has mitigated the health, and economic impacts of the COVID-19 pandemic on wildlife conservation.

Population, Health and Environment (PHE) is an integrated community-based approach to development that acknowledges and addresses the complex connections between humans, their health, and the environment (25). Planetary health recognizes the effects of human behavior on the environment, which in turn has an impact on human health (26). One Health has a strong emphasis on biosurveillance and biosecurity of farm animals and wildlife where, measures taken often involve the culling of animals in order to prevent the spread of diseases to humans. However, for better mitigation and prevention of epidemics, it is necessary to adopt a more than human approach, that also emphasizes the welfare of animals (27). Non-human primates, and gorillas in particular are emblematic species to show the value of a One Health approach that equally addresses the health of humans, animals, and their ecosystems.

## Mitigating the Impact of COVID-19 on Great Ape Health and Community Well-Being

### Reducing the Threat of Disease Transmission From People to Gorillas

There have been a number of incidences of human respiratory virus transmission from humans to wild great apes that originated from local communities or tourists (28–30). Owing to the potential for reverse zoonosis, guidelines to minimize disease transmission between people and gorillas were instituted by the government agency responsible for wildlife, Uganda Wildlife Authority (UWA) with support from International Gorilla Conservation Programme (IGCP) when tourism began in 1993 (www.igcp.org). These rules included not being allowed to visit the gorillas when showing signs of illness, maintaining a 5-m distance and turning away to cough or sneeze. Over the past two decades as the gorillas became more habituated to the presence of humans, the viewing distance between people and gorillas became closer than the new 7-m viewing distance, where research revealed that 60% of the time the tourists got closer than 3 m and 40% of the time, it was the gorillas that got closer than 3 m (31). Research studies also revealed that 51–73% of tourists were willing to wear masks (31, 32) to minimize the spread of respiratory diseases to gorillas.

To minimize the risk of COVID-19 and other respiratory diseases spreading from people to gorillas, CTPH worked with UWA and conservation and health NGOs including IGCP, Mountain Gorilla Veterinary Project/Gorilla Doctors, Max Planck Institute and Bwindi Community Hospital (BCH) to train 400 park staff to prevent transmission of diseases between people and from people to gorillas. Park staff who monitor the health of gorillas and protect them through law enforcement patrols in the forest, were trained to put on protective face masks, enforce hand hygiene, and a 7-m great ape viewing distance, which UWA increased to 10 m as an additional measure during the pandemic (33). The rangers were also provided with double layered cloth face masks and hand sanitizers. A new regulation was instituted to have mandatory temperature checks using a non-contact infrared thermometer, for every person entering the forest who also had to wash their hands and disinfect their boots before and during the trek to the gorillas.

The same training was given by CTPH, UWA, BCH, and Kanungu and Kisoro Districts Health Offices to 119 Gorilla Guardians who are community volunteers from the Human and Gorilla Conflict Resolution (HUGO) team, supported by UWA, IGCP, and CTPH to herd gorillas from community land to the park, which occurs a few days every month among habituated gorilla groups at Bwindi and to monitor their health when in community land (34). The Gorilla Guardians were also given double layered cloth face masks, hand sanitizers and liquid soap. CTPH, IGCP and The Gorilla Organization donated double layered cloth face masks and non-contact infrared thermometers to UWA and the Gorilla Guardians. Village Health and Conservation Teams (VHCTs) who are community health volunteers, trained by CTPH since facilitating their formation in 2007, to conduct behavior change communication at the household and village level on good hygiene and sanitation, infectious disease prevention and control, family planning, nutrition, sustainable agriculture, gorilla, and forest conservation as well as report homes visited by gorillas were also trained by CTPH, BCH, Kanungu and Kisoro District Health Office staff and UWA, to mitigate COVID-19, which resulted in an increase in hand washing facilities at their homes because of the fear of contracting the disease. Tuberculosis and other respiratory diseases were managed together with COVID-19 where people presenting with cough, flu, and difficulty in breathing were tested for both diseases. Since the pandemic began, the VHCTs have reached over 5,000 households with critical health and conservation information and services. The 270 VHCTs were also trained to identify suspects and carry out contact tracing as well as counsel confirmed cases and their contacts. Gorilla Guardians and VHCTs were also given posters on preventing COVID-19 between people and from people to gorillas to disseminate in their community, which were made for CTPH by Solidaridad, a donor of CTPH. Over 500 posters were put at the park offices and disseminated among the local communities.

Conservation Through Public Health is working with UWA, BCH, Kanungu and Kisoro District Health offices, Uganda Virus Research Institute, University of Madison-Wisconsin, and other partners to test gorillas, and people interfacing with gorillas both inside and outside the park for COVID-19. During the pandemic, CTPH got new donors to fund these activities as part of emergency funding for COVID-19, including Arcus Foundation, International Union for the Conservation of Nature (IUCN) Save the Species and European Union, the British High Commission and individual donors, which was complimented by funding for ongoing activities supported by Tusk Trust, Whitley Fund for Nature, Population Connection and Wildlife Conservation Network.

Conservation Through Public Health (CTPH) joined the Uganda Ministry of Health (MOH) national disease taskforce in 2010. Through this platform, CTPH increased awareness among taskforce members of the susceptibility of gorillas and chimpanzees to COVID-19 from humans, influencing the national response to the pandemic where primate tourism reopened later than other wildlife based tourism primarily to protect the closely genetically related gorillas and chimpanzees from human diseases as stated in the speech of the President of Uganda in May 2020 (www.ntv.co.ug).

### Reducing the Threat of Poaching

The absence of tourism income also became a threat to the survival of the mountain gorillas because the economic incentive for communities to protect them by not entering their habitat to poach was removed. Additionally, there was a reduction in tourism revenue that sustains park operations including anti-poaching patrols. This prompted the government to reopen primate tourism at the end of September 2020 where the benefits of reducing illegal entries in the habitat of great apes outweighed the risk of introducing COVID-19 from tourists to the endangered gorillas and chimpanzees because the risk of reverse zoonotic disease transmission was perceived to be greatly reduced through instituting the new standard operating procedures including mandatory wearing of masks within 10 m of great apes and increasing the viewing distance from seven to 10 m. Vaccination of people who interact with great apes potentially reduce the risk of COVID-19 disease transmission between people and great apes even further (35). Though tourism is still at an estimated 10–20% of pre pandemic levels largely due to lockdowns preventing international travel, it has brought hope to the Bwindi local communities and contributed to a reduction in poaching, as well as, generated revenue to support law enforcement operations that protect the gorillas and other species in their habitats.

When the COVID-19 pandemic reached Uganda in March 2020, double layered cloth face masks were bought from a local enterprise, Ride for a Woman, and provided to park rangers and other conservation personnel and community members including HUGOs, VHCTs and reformed poachers. This provided an income for the women and contributed to a reduction in poaching in the gorillas' habitat during the pandemic that resulted in a reduction in tourism due to global lockdowns.

In May 2020, CTPH got a new UK-based distributor, Moneyrow Beans, for its Gorilla Conservation Coffee social enterprise that had started in 2015 to provide above market prices for premium and specialty coffee sold locally and internationally to Lifestyle of Health and Sustainability (LOHAS) consumers who want to support gorilla conservation where a donation from every bag of coffee sold goes toward sustaining community health, gorilla health, and conservation education programs of CTPH. This social enterprise also had reduced sales because it relied on international tourists who could no longer travel to Uganda for tourism to the gorillas and other wildlife and who were the main customers for the coffee. Finding a new export market not dependent on tourism enabled 150 coffee farmers to earn some revenue and reduced their need to poach. It also enabled conscious consumers to fulfill their desire of supporting gorilla conservation during the pandemic through purchase of coffee in the UK, USA, Kenya, New Zealand and Australia.

As a response to the killing of Rafiki, the lead silverback of Nkuringo gorilla group by a hungry poacher, that led to the group reducing in size, CTPH started a new emergency food relief “Ready to Grow” program to provide fast growing seedlings that take 1–4 months to produce food, to reduce hunger in vulnerable community members where 1,002 families were the first to be provided with 10 types of fast growing seedlings helping to reduce hunger, starting with Nteko parish where the family of the poacher who speared Rafiki the gorilla were also beneficiaries. Among the vulnerable people who received seedlings were porters whose livelihoods were most affected by the lack of tourism, which they had began to solely depend on to meet their family needs, reformed poachers who CTPH had started to provide with group livestock income generating projects, Batwa hunter gatherers who were resettled outside the park when it was gazetted in 1991, Gorilla Guardians, local council chairpersons, and VHCTs who were also tasked with monitoring the success of the Ready to Grow program among the households. CTPH is currently measuring how improving the well-being of Bwindi local communities is reducing poaching and other illegal forest resource use during the pandemic.

### Advocating for Responsible Tourism to Great Apes Through a One Health Approach

In March 2020, a network of African based NGOs and CBOs was created to strengthen the African voice in the Convention of Biological Diversity (CBD) resulting in the creation of the Africa CSO Biodiversity Alliance (ACBA) of which CTPH became a member. Through the ACBA platform CTPH worked with IGCP to develop a policy brief targeting African governments, donors and tour companies, (36) based on IUCN best practice guidelines for great ape tourism and lessons learned from 27 years of implementing great ape tourism in Uganda, which prior to the COVID-19 pandemic, was contributing to 60% of tourism revenue for UWA (37). Through the policy brief and the MOH COVID-19 taskforce, CTPH has been advocating for priority testing and vaccination of park staff coming into close contact with gorillas and chimpanzees.

In response to the pandemic, the ACBA designed a social media campaign on the risks of zoonosis to human health (38) and reverse zoonosis to great apes, with links to COVID-19, highlighting the danger of consuming bats, primates, and other high risk species, that have historically been the source of disease outbreaks and epidemics of Ebola, Marburg, and other zoonotic diseases in Africa and the risks of spreading diseases from people to closely related great apes (39, 40).

## Discussion

A One Health approach that equally addresses the health of humans and gorillas together to promote species and habitat conservation enabled CTPH to mitigate the impact of COVID-19 on gorilla conservation. Lessons from the COVID-19 pandemic in Uganda that can be applied to other countries in Africa and the developing world even after the pandemic has been brought under control, include the great need to prevent COVID-19 and other diseases between people and non-human great apes through responsible tourism and promotion of community health where great apes and other wild animals range. Another lesson is the need to reduce poaching by addressing hunger among vulnerable community members and by supporting community livelihoods. Tourism is one of the most effective ways of providing sustainable financing for conservation, however it must be carried out carefully to minimize the risk of zoonotic disease transmission to great apes from tourists who could bring in fatal viruses like SARS-COV-2. In order for tourism to contribute to a reduction in poaching from local communities, it must support local livelihoods. The pandemic has also demonstrated the need to provide other sustainable financing mechanisms for conservation when tourism is not possible. This includes increasing access to international markets by encouraging responsible consumption through purchase of ethically sourced products that improve community livelihoods with a direct positive impact on conservation, such as Gorilla Conservation Coffee. The delicate balance between the increased risks to the health of great apes from tourists and increased poaching in their habitat due to the absence of tourism has to continually be assessed to determine whether to suspend or reopen great ape tourism during pandemics (17).

Thus, there is a critical need for One Health approaches that improve human, animal and ecosystem health together and support communities through tourism and livelihoods that are not dependent on tourism. Such approaches can be scaled globally to build resilience and minimize the health and economic impact of pandemics like COVID-19 on humanity and wildlife particularly in low to middle income countries (41, 42).

## Data Availability Statement

The datasets presented in this study can be found in online repositories. The names of the repository/repositories and accession number(s) can be found at: www.ctph.org.

## Author Contributions

GK-Z, SR, and LZ designed the programs. AN led the implementation of the field programs. GK-Z led advocacy initiatives and wrote the manuscript. GK-Z, SR, and LZ participated in implementation of the activities. All authors contributed to the article and approved the submitted version.

## Conflict of Interest

The authors declare that the research was conducted in the absence of any commercial or financial relationships that could be construed as a potential conflict of interest.

## Publisher's Note

All claims expressed in this article are solely those of the authors and do not necessarily represent those of their affiliated organizations, or those of the publisher, the editors and the reviewers. Any product that may be evaluated in this article, or claim that may be made by its manufacturer, is not guaranteed or endorsed by the publisher.
